# Untargeted Metabolomics Approach for the Discovery of Salinity-Related Alkaloids in a Stony Coral-Derived Fungus *Aspergillus terreus*

**DOI:** 10.3390/ijms251910544

**Published:** 2024-09-30

**Authors:** Yayue Liu, Li Wang, Yunkai Feng, Qingnan Liao, Xiaoling Lei, Xueqiong Hu, Longjian Zhou, Yi Zhang

**Affiliations:** 1 Guangdong Provincial Key Laboratory of Aquatic Product Processing and Safety, Guangdong Provincial Engineering Laboratory for Marine Biological Products, Guangdong Provincial Center for Modern Agricultural Scientific Innovation, Shenzhen Institute of Guangdong Ocean University, Zhanjiang Municipal Key Laboratory of Marine Drugsand Nutrition for Brain Health, Research Institute for Marine Drugs and Nutrition, College of Food Science and Technology, Guangdong Ocean University, Zhanjiang 524088, China; yayue_liu@163.com (Y.L.); wangli991003@163.com (L.W.); 13582945747@163.com (Y.F.); 17854225126@163.com (Q.L.); leixl-19@126.com (X.L.); hxw247@163.com (X.H.); 2 Southern Marine Science and Engineering Guangdong Laboratory (Zhanjiang), Zhanjiang 524088, China; 3 Collaborative Innovation Center of Seafood Deep Processing, Dalian Polytechnic University, Dalian 116034, China

**Keywords:** untargeted metabolomics, stony coral, *Aspergillus terreus*, alkaloids

## Abstract

As a part of the important species that form coral reef ecosystems, stony corals have become a potential source of pharmacologically active lead compounds for an increasing number of compounds with novel chemical structures and strong biological activity. In this study, the secondary metabolites and biological activities are reported for *Aspergillus terreus* C21-1, an epiphytic fungus acquired from *Porites pukoensis* collected from Xuwen Coral Reef Nature Reserve, China. This strain was cultured in potato dextrose broth (PDB) media and rice media with different salinities based on the OSMAC strategy. The mycelial morphology and high-performance thin layer chromatographic (HPTLC) fingerprints of the fermentation extracts together with bioautography were recorded. Furthermore, an untargeted metabolomics study was performed using principal component analysis (PCA), orthogonal projection to latent structure discriminant analysis (O-PLSDA), and feature-based molecular networking (FBMN) to analyze their secondary metabolite variations. The comprehensive results revealed that the metabolite expression in *A. terreus* C21-1 differed significantly between liquid and solid media. The metabolites produced in liquid medium were more diverse but less numerous compared to those in solid medium. Meanwhile, the mycelial morphology underwent significant changes with increasing salinity under PDB cultivation conditions, especially in PDB with 10% salinity. Untargeted metabolomics revealed significant differences between PDB with 10% salinity and other media, as well as between liquid and solid media. FBMN analysis indicated that alkaloids, which might be produced under high salt stress, contributed largely to the differences. The biological activities results showed that six groups of crude extracts exhibited acetylcholinesterase (AChE) inhibitory activities, along with 1,1-diphenyl-2-picrylhydrazyl (DPPH) free radical scavenging and antibacterial activities. The results of this study showed that the increase in salinity favored the production of unique alkaloid compounds by *A. terreus* C21-1.

## 1. Introduction

As one of the earliest organisms studied in the field of marine drugs, coral contains abundant symbiotic microbial resources and has shown promising potential as a source of pharmacologically active lead compounds due to the chemical diversity of its secondary metabolites [1,2,3]. Notably, fungi have played an important role in the identification of numerous lead compounds, exhibiting remarkable biological activities including anti-inflammatory [4], antibacterial [5], cytotoxic [6], antiviral [7], and enzyme-inhibitory properties [8]. It has been reported that over 486 new natural products, including polyketides, terpenoids, alkaloids, peptides, and other compounds, have been discovered from coral-associated fungi in the past decade [9,10]. These compounds were obtained from different types of coral-derived fungi belonging to 31 genera, involving *Cladosporium* sp., *Penicillium* sp., *Chondrostereum* sp., *Sarcophyton* sp., and *Aspergillus* sp. *Aspergillus* sp., accounting for 26% of the production of new natural products, is a typical coral-associated fungus, and usually produces terpenes, alkaloids, peptides, etc. For example, three new indole diketopiperazine alkaloids named 11-methylneoechinulin E, variecolorin M, and (+)-variecolorin G, together with a known compound, (-)-variecolorin G, were isolated from a soft coral-associated epiphytic fungus *Aspergillus* sp. EGF 15-0-3 [11]. Cheng et al. obtained six new polycyclic alkaloids, namely versiquinazolines A, B, G, and K and versiquinazolines L–Q from gorgonian-derived fungus *Aspergillus versicolor* [12]. However, the current studies on coral symbionts or epiphytes predominantly focus on specific coral families such as gorgonacea or soft corals, accounting for a significant majority of 76%. Conversely, there is a dearth of reports regarding the exploration of active natural compounds from other coral families, particularly stony corals and their associated fungi, representing only 6% [1,3].

Fungi contain a significant number of biosynthetic gene clusters (BGCs) for secondary metabolites. However, a large portion of fungal BGCs remain inactive under laboratory or artificial culture conditions [13]. In recent years, various innovative approaches have been developed to activate cryptic gene clusters for expression, and the “One Strain, Many Compounds” (OSMAC) method has gained increased attention from researchers due to its ability to enhance the diversity of fungal metabolites, which is achieved through manipulation of medium composition, optimization of fermentation conditions, or introduction of small molecules [14,15,16]. By utilizing OSMAC technology, numerous bioactive natural products have been successfully discovered [17,18,19,20,21,22]. For instance, the novel pentacyclic indole alkaloids asperinamide B and peniochroloid B, exhibiting anti-FADU (Human pharyngeal squamous cell) potential, were extracted from the endophytic fungus *Penicillium oxalicum* 2021CDF-3 utilizing the OSMAC strategy [15]. Furthermore, four uncommon phenylhydrazine alkaloids known as talarohydrazones A–D were isolated from the deep-sea cold seep-derived fungus *Talaromyces amestolkiae* HDN21-0307 using the OSMAC method and MS/MS-based molecular networking (MN) technology [20].

During our ongoing search for novel marine natural products [23,24,25], a fungus *A. terreus* strain C21-1 was acquired from a stony coral, *Porites pukoensis*, collected from Xuwen Coral Reef Nature Reserve in China. In this study, we present a metabolomic investigation of this strain, with a focus on the influence of cultural conditions. Modifications of culture conditions were performed using six different media including PDB and rice medium (of varying types) following the OSMAC approach to evaluate their impact on alkaloid production, particularly the effects of salinity. The culture extracts were subjected to untargeted metabolomics analysis using FBMN to assess variations in secondary metabolite profiles. Additionally, crude extracts were evaluated for their AChE inhibitory, DPPH free radical scavenging, and antibacterial activities.

## 2. Results

### 2.1. Morphological Comparison

**Figure 1:**
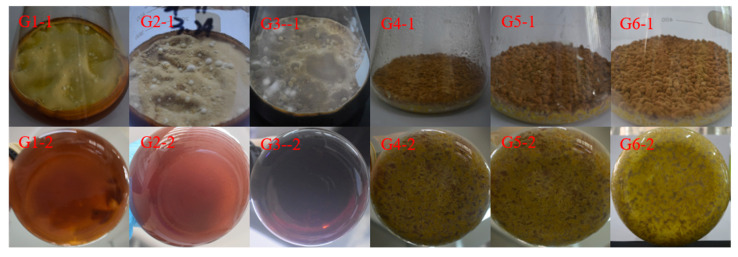
The morphological characteristics of *Aspergillus terreus* C21-1 in different media over a period of 28 days (G1–G6); 1 means the front and 2 the back of the plate.

*A. terreus* C21-1 was cultivated at room temperature for 28 days in PDB and rice media under six experimental condition groups, namely, (G1) 0.3% salinity PDB (abbreviated as 0.3 PDB), (G2) 3% salinity PDB (abbreviated as 3 PDB), (G3) 10% salinity PDB (abbreviated as 10 PDB), (G4) 0.3% salinity brown rice medium (abbreviated as 0.3 BRM), (G5) 0.3% salinity fragrant rice medium (abbreviated as 0.3 FRM), and (G6) 0.3% salinity northeast rice medium (abbreviated as 0.3 NRM). The results depicted in Figure 1 demonstrate significant variations in the colony morphology of *A. terreus* C21-1 under different salinity conditions. In the presence of 0.3 PDB media, bright yellow colonies and a yellowish-brown bacterial solution were observed (Figure 1). However, as salinity increased, the mycelia became thinner and the liquid turned brown, indicating potential differences in secondary metabolite production due to varying salinity levels. Conversely, when grown on rice medium, all strains exhibited brown colonies with no significant changes in colony morphology across different rice species, which indicated that the strain’s secondary metabolites were minimally influenced by the rice variety.

### 2.2. Comparison of TLC Fingerprints and HPLC

**Figure 2:**
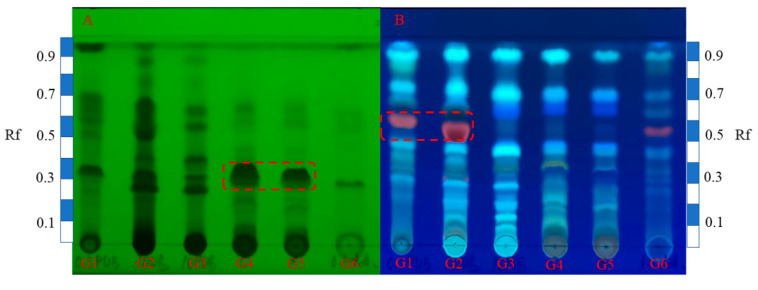
HPTLC fingerprints of six crude extracts are presented in the following figures. (**A**) shows the UV images of experiments G1–G6 under 254 nm, with sample numbers marked below the starting line. (**B**) displays the UV images of G1–G6 under 365 nm. The unfolding system used was *n*-hexane: ethyl acetate = 3:2 (*v*/*v*). The rulers beside the TLC plate were utilized as references for calculating the Rf values.

The secondary metabolite compositions in TLC fingerprints were observed at 254 nm (Figure 2A) and 365 nm (Figure 2B) for comparative analysis. Under the wavelength of 254 nm, a significant increase in the main products (*R*_f_ = 0.3) of G4 and G5 was observed in the rice medium compared to G6. Although there were some variations in other trace components, the primary products of different rice varieties remained consistent. At 365 nm, only G6 exhibited red spots in the rice medium at the *R*_f_ value of 0.5, while the other products remained unchanged. In contrast, within the PDB medium at 254 nm, discernible differences were noted among G1, G2, and G3, with significantly higher abundance of metabolites observed for G2 and G3 compared to G1. At 365 nm, red fluorescent spots (*R*_f_ = 0.5) were detected in both PDB media for both G1 and G2 but not for G3, whose production seemed to decrease with increasing salinity levels. Overall, metabolites within the PDB medium exhibited significantly higher abundance than those present in the rice medium. Furthermore, an increase in salinity within the PDB medium resulted in notable differences among strains’ metabolites.

**Figure 3:**
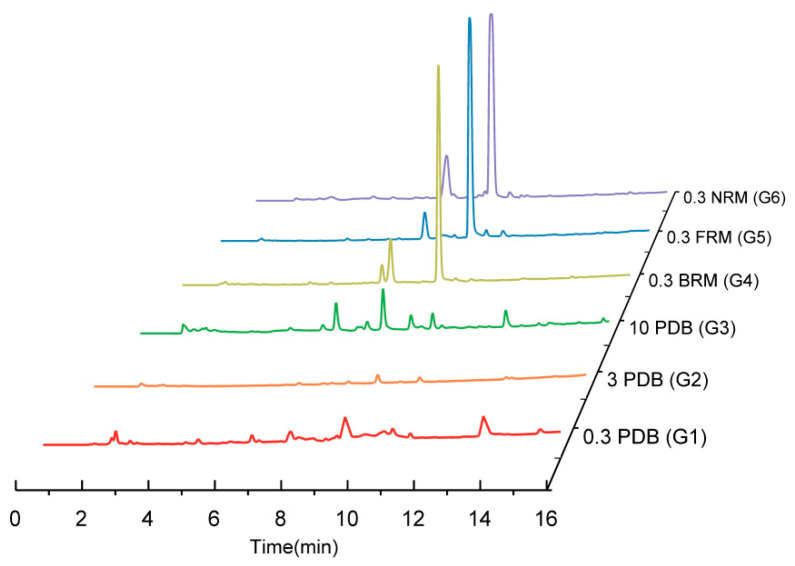
The HPLC fingerprints of the six crude extracts using a UV wave length of 210 nm.

Further HPLC analysis revealed distinct variations in the number of chromatographic peaks and peak area sizes within the crude extracts obtained from G1 to G6, as shown in Figure 3. Firstly, a common chromatographic peak at t_R_ = 9.1 min was consistently observed across all six media, serving as the predominant product in all cases except for G3. Secondly, minimal differences were observed in the HPLC profiles of secondary metabolites among G4–G6 cultivated on rice media, which aligned with the earlier findings from strain morphology and TLC analysis. In contrast, significant disparities were detected among G1–G3 grown in the PDB medium. Notably, an increase in chromatographic peaks indicated enhanced abundance on secondary metabolites. Additionally, there was a notable reduction in peak area corresponding to the main product’s chromatographic peak, suggesting decreased yield. Lastly, compared to G1 and G2, G3 exhibited a greater number of chromatographic peaks with varying retention times, implying its composition includes diverse metabolites.

### 2.3. Biological Activities of Crude Extracts

The crude extracts were subjected to testing for their inhibitory activities against AChE and DPPH free radical scavenging and antibacterial properties. The crude extracts of G1, G4, G5, and G6 showed good AChE inhibitory activity, with inhibition rates greater than 70% (Table 1). The IC_50_ values of G4, G5, and G6 were close to those of the positive control (IC_50_ of parnepezil hydrochloride = 0.2 μg/mL). Under the condition of high salinity, the activity of G2 and G3 decreased, which indicated that G1, G4, G5, and G6 crude extracts had more products to inhibit AChE activity. Moreover, all components showed significant DPPH free radical scavenging activities at the concentration of 0.5 mg/mL, with an inhibition rate surpassing 50.0%. Among them, G1, G4, G5, and G6 exhibited stronger scavenging activities with an inhibition exceeding 90.0%, albeit slightly weaker than the positive control (Table 2). The results of DPPH radical scavenging and AChE inhibition remained consistent, with activity decreasing with increasing salinity. Regarding antibacterial activities against six microorganisms (Table 3), G1 exhibited notable antibacterial effects, along with G4 and G5, specifically against Methicillin-resistant *Staphylococcus aureus* (MRSA) bacterial strains, which had measured inhibition zone diameters of approximately 14.0 ± 0.6, 10.5 ± 0.2, and 14.0 ± 0.6 mm, respectively. These values were comparable to those observed in the positive control (14.8 ± 0.8 mm). Furthermore, G5 also demonstrated a strong inhibitory effect on *Vibrio alginolyticus*, with an inhibition zone diameter measuring 18.0 ± 0.5 mm, approaching that observed in the positive control (19.0 ± 0.6 mm). However, G2, G3, and G6 showed no or weak inhibitory effects on any of these six bacterial strains, whose measured inhibition zone diameters were less than 10.0 mm.

### 2.4. LC-MS/MS Based Data Statistical Analysis

**Figure 4:**
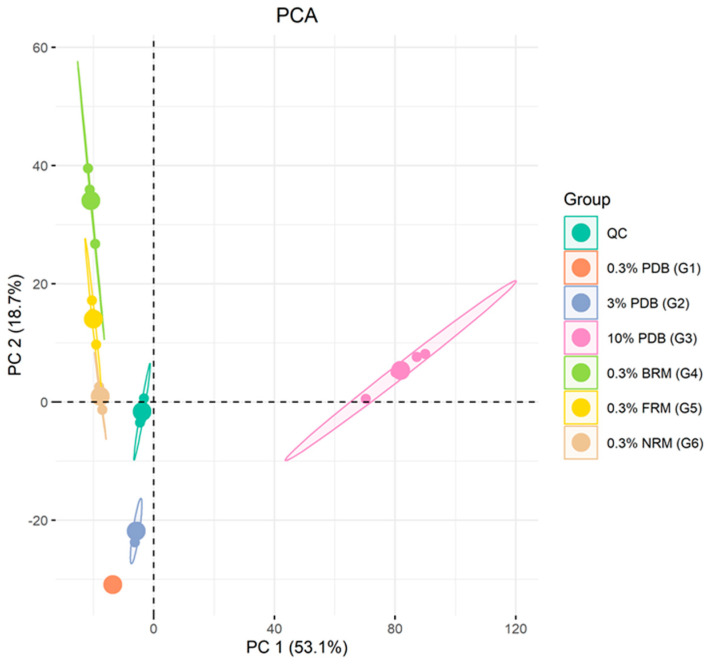
PCA analysis of all groups of the *A. terreus* C21-1.

The secondary metabolite variations were further analyzed using untargeted metabolomics studies. After fermentation and extraction, the chemical characteristics of G1–G6 were examined using LC-MS/MS in positive ion mode. The resulting data were transformed into a matrix using automatic peak detection and subjected to multivariate data analysis for comparative assessment. To ensure reliability, each culture was independently replicated four times. The PCA score plot (Figure 4) for all 24 samples revealed significant disparities in their chemical characteristics. The clustering and dispersion patterns observed in the PCA plot indicate the similarity of metabolic fractions between samples, with closer proximity indicating higher similarity and greater distance implying dissimilarity [26,27]. In this plot, PC1 = 53.1% represented the horizontal coordinate, denoting scores of the first-ranked principal component, while PC2 = 18.7% represented the vertical coordinate, denoting scores of the second-ranked principal component. G1 and G2 exhibited close similarity as they clustered together, suggesting comparable secondary metabolite profiles between them. Likewise, G4, G5, and G6 displayed similar secondary metabolite profiles based on their aggregation pattern in the PCA plot. The products obtained from high-salinity culture conditions under 10% PDB (G3) medium showed distinct separation from those produced under different conditions, suggesting significant differentiation in their secondary metabolite production under high-salinity culture conditions compared to those produced under different conditions.

**Figure 5:**
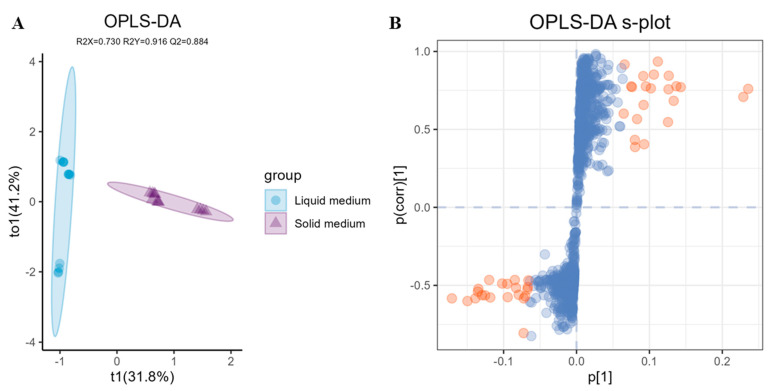
(**A**) OPLS-DA plot (R2X: 0.730, R2Y:0.916, Q2: 0.884) of liquid medium and solid medium groups of the *A. terreus* C21-1 metabolic profiles. (**B**) OPLS−DA s−plot for liquid medium vs. solid medium groups. Dots represent individual metabolites; dots highlighted in light orange correspond to metabolites with a VIP value greater than 3.

A comparative analysis was conducted using OPLS-DA to elucidate the disparities in metabolite distribution between liquid and solid medium extracts. The results (Figure 5A) revealed significant differences in the first dimension between the two groups, with solid medium extracts showing reduced dispersion in orthogonal components compared to their liquid medium counterparts. This distinction was particularly evident for the 10 PDB profiles, which formed a distinct cluster separated from other PDB extracts. The OPLS-DA model parameters RX2, R2Y, and Q2 were 0.730, 0.916, and 0.884, respectively, indicating a robust OPLS-DA model with good reliability and predictive ability. The OPLS-DA s-plot depicted in Figure 5B highlights the unique metabolites that distinguished these two groups. Features exhibiting a significant variable importance in projection (VIP) values over 1 were considered as contributing features, with their contribution to component differences increasing as VIP values increased. In this study, metabolites with VIP values over 3 were selected and identified through comparison using MS-Finder (Table S1). Unfortunately, several annotations could not be assigned due to either insufficient data within existing databases or because they represent novel compounds.

**Figure 6:**
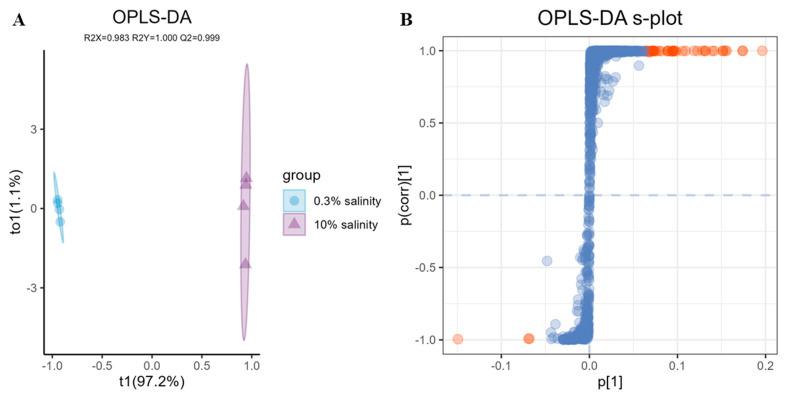
(**A**) OPLS−DA plot (R2X: 0.983, R2Y:1.000, Q2: 0.999) of 0.3% salinity and 10% salinity groups. (**B**) OPLS−DA s−plot for 0.3% salinity vs. 10% salinity groups. Dots represent individual metabolites; dots highlighted in light orange correspond to metabolites with a VIP value greater than 3.

To accurately assess the specific impact of salinity on the induction of *A. terreus* C21-1 metabolites, an OPLS-DA comparison was further conducted between 0.3% salinity and 10% salinity (Figure 6A). The validated OPLS-DA model revealed significant disparities between these two groups, with the 10% salinity fraction displaying noticeable deviation from other fractions. This observation suggested that a salinity level of 10% differs significantly from that of 0.3%. Furthermore, the OPLS-DA model’s robustness, as indicated by its parameters (R2X, R2Y, and Q2), confirmed the model’s reliability and predictive ability, supporting the results. Metabolites with VIP > 3 in the OPLS-DA s-plot were selected for comparison and identification using MS-Finder. The results (Table S2) demonstrated that the majority of metabolites with VIP > 3 under both low- and high-salinity conditions were alkaloids.

### 2.5. LC-MS/MS-Based MSDIAL Analysis and FBMN Networking

**Figure 7:**
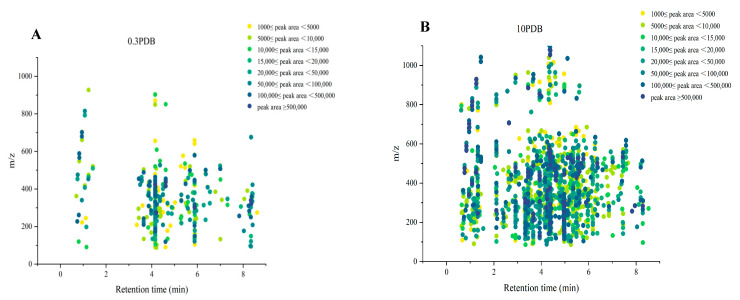
The metabolic profile of the features in extracts 0.3 PDB (**A**) and 10 PDB (**B**) showing their retention times, precursor ion *m*/*z* values, and intensities (exported from MSDIAL).

**Figure 8:**
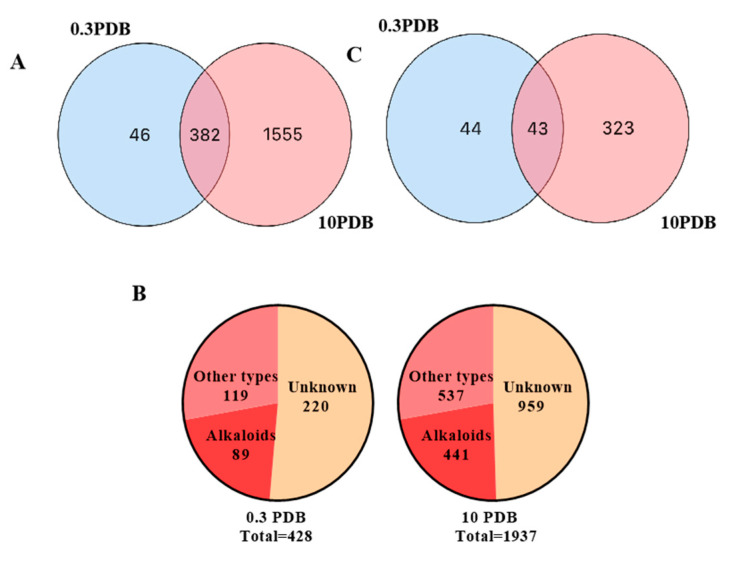
The statistics of the numbers of the total features (**A**), the annotated and unknown features (**B**), and the alkaloids (**C**) detected in the two crude extracts.

To investigate the diversity of alkaloids, the extract samples 0.3 PDB and 10 PDB were further analyzed by LC-MS/MS and successive multi-approach-assisted FBMN networking. Firstly, MSDIAL-generated 2D peak spot heatmaps (Figure 7) display the metabolic profiles of the two samples, including precursor *m*/*z* values, retention times, and peak areas of all features. These features were primarily annotated by matching the internally loaded database. In total, 1555 features were successfully identified by MSDIAL, comprising 428 from the 0.3 PDB condition, 1937 from the 10 PDB condition, and an overlap of 382 features between both conditions (Figure 8A).

FBMN enables the generation of molecular networks from mass spectrometry data, which facilitates isomer identification, relative quantification incorporation, and integration of ion mobility data [28,29]. This workflow allows users to import quantitative information from feature detection tools into molecular networks, and thus differentiate isomers based on retention times within the network [30]. The formula and MS2 information obtained from FBMN constructed by LC-MS/MS can also be utilized for automated metabolite annotation using MSDIAL [31] and MSFINDER [32], which search in multiple databases through formula matching and comparison between measured MS/MS spectra and reference MS/MS spectra [33]. Through the comprehensive compound annotation strategy of multi-approach-assisted FBMN [34], it was determined that there was a total of 428 feature points in 0.3 PDB, out of which 208 could be annotated, while the remaining 220 remained unknown. Among these feature points that could be annotated, a significant number, of 106 (42.8%), were identified as alkaloids or nitrogen-containing compounds. Similarly, in the case of 10 PDBs, there existed a total of 1937 feature points, with 978 able to be annotated and the remaining 959 still unknown. Out of these feature points that could be annotated, a substantial number, of 441 (45.1%), were classified as alkaloids (Figure 8B). As depicted in Figure 8C, both datasets contained alkaloids that could be annotated, with counts of 89 and 441 for the respective datasets. Furthermore, there were also overlapping annotations, with a count of exactly 43 alkaloids common to both datasets. Notably, compared to the medium containing the 0.3 PDB dataset, the medium containing the 10 PDB dataset exhibited enhanced capability for metabolizing alkaloid production.

**Figure 9:**
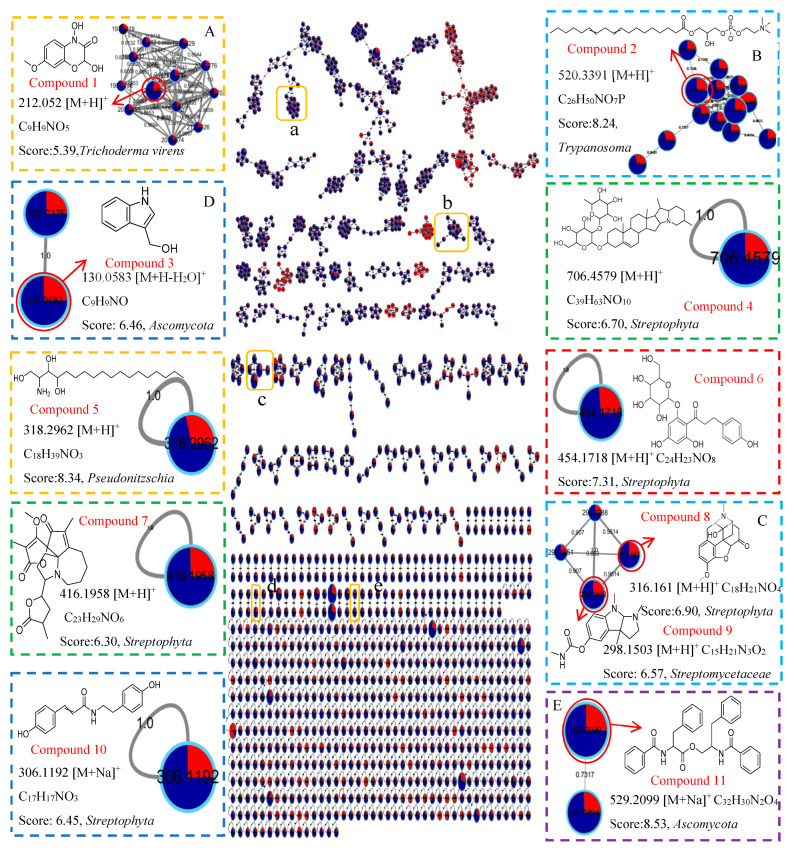
The FBMN molecular network based on positive ion MS/MS spectral similarity, showing a selection of amplified clusters. In the FBMN network, each node represents a feature marked with the mean *m*/*z* value of the parent ion, and the similarity of the secondary mass spectra between compounds was expressed by the cosine value, which was proportional to the similarity. The different colors of sections in the nodes represent different samples, i.e.,: 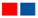 respectively, 0.3 PDB and 10 PDB. The thickness of the connecting lines between nodes is positively correlated with the cosine value. The node size reflects the feature abundance (ion intensity). The A, B, C, D, E represent a locally enlarged view of a, b, c, d, e, respectively.

Molecular network maps were constructed using the FBMN method based on 0.3 PDB and 10 PDB crude extracts (Figure 9). Compounds with significant differences between the 10 PDB and 0.3 PDB extracts were identified through a cross-node search, and then mapped to the MN. Among the nodes corresponding to VIP > 3 (Table S2), the structure of 11 alkaloids can be determined or inferred (Figure 9). Compound **1** was annotated as DIMBOA [35]. DIMBOA has been claimed to be a natural defense agent against bacteria, fungi, and insects [36]. Furthermore, benzoxazinones and some of their derivatives have been utilized in pharmaceutical development [37]. Compound **9** was identified as physostigmine ([(3a*R*, 8b*S*)-3,4,8b-trimethyl-2,3a-dihydro-1H-pyrrolo [2,3-b]indol-7-yl] N-methylcarbamate), known as eserine. It acts as a tertiary amine that reversibly inhibits AChE [38], showing promise in mitigating AD-associated pathologies [39].

## 3. Discussion

It has been confirmed that there is a large number of genes encoding compounds in fungi. However, the actual number of compounds isolated from fungi is much lower than the number of compounds encoded by these genes, indicating that many of these genes are in a silent state. Activating these silent genes would enable microorganisms to express new secondary metabolites and increase the chemical diversity of microorganisms. One effective method able to activate silent gene expression is OSMAC, which offers advantages such as low cost, high efficiency, and easy operation. In some cases, fungal responses to salinity stress are implicated in the biosynthesis of certain metabolites [40]. To adapt to a high-salinity environment, microorganisms must employ mechanisms to maintain intracellular osmotic pressure in equilibrium with the external medium [41]. Fungi have the ability to detect changes in osmotic pressure from their external environment, which can lead to the activation of transcription factors through various cellular signaling pathways. In this study, we observed that exposure to a 10 PDB medium (with 10% salinity) resulted in the overexpression of certain alkaloids in the fungus. This suggests that high-salt conditions may induce a series of changes in the fungus, ultimately leading to the overexpression of specific alkaloids.

Untargeted metabolomics involves the analysis of the metabolic state of biological systems by measuring the concentration profiles of all measurable free low-molecular-weight metabolites [42]. Mass spectrometry (MS)-based metabolomics is increasingly important in facilitating natural product research. In their study, Le et al. utilized PCA, OPLS-DA, and FBMN to conduct an untargeted metabolomics analysis of *Penicillium restrictum* MMS417. The results revealed that the addition of mussel extract to the strain led to the production of various compounds, including sterols, macrolides, and pyran-2-ones [43]. Kang et al. analyzed the secondary metabolites of seven species of *Trichoderma* (33 strains) using its sequence and metabolome-based chemotaxonomic comparison. They found that the chemical taxonomy based on secondary metabolites was more accurate than its sequence and identified an unknown group of *Trichoderma* [44]. Chen used HPLC fingerprinting combined with stoichiometric analysis of *Ganoderma lucidum* fruiting bodies and screened four marker components as discriminative variables to distinguish *Ganoderma lucidum* [45]. In this experiment, we not only analyzed the differences in the production and types of secondary metabolites in different media by TLC and HPLC, but also conducted non-targeted metabolomics analysis. PCA and OPLS-DA analysis were performed using LC-MS/MS data, and the differences in secondary metabolites were visualized using graphing software. By locking in the VIP values, the samples showed some compounds with significant differences. In addition, we used FBMN technology to identify these compounds.

It has been reported that halotolerant fungi have special osmoregulatory mechanisms to regulate intracellular osmotic potential, which allows them to survive under a variety of saline conditions, including high-salt conditions [46]. The secondary metabolites produced by the organism may be regulated by environmental factors such as salinity [47]. The membrane structure of halophilic microorganisms plays a crucial role in their adaptation to saline conditions. It serves to protect the cell from the harmful effects of salt concentrations changing and maintains osmotic homeostasis by regulating membrane fluidity [48]. Structural modifications, as well as the presence of pigments and/or hydrophobins, are observed in both the plasma membrane and the cell wall [49,50,51]. High salinity leads to an increase in cell wall thickness and influences changes in lipid composition, including alterations in sterol quantity, fatty acyl chain type, and polar phospholipid head-group nature [52]. However, limited research has been conducted on the self-adaptation of fungi in high-salinity conditions. The study by Ding et al. utilized CLUSTER Profiler (v3.4.4) to elucidate the biochemical pathway of differentially expressed genes (DEGs) through KEGG mapping. They identified 43 significant DEGs associated with the biosynthesis of amino acids and other nitrogen-containing compounds [53]. DEGs are abundant in alanine, aspartate, and glutamate metabolism, as well as the ornithine cycle. Therefore, it was concluded that the accumulation of amino acids or their derivatives plays a crucial protective role in the adaptation of halophilic fungi to high salinity. Among these, biuret (a product of the ornithine cycle) was significantly accumulated in high-salt-induced *Monteverde* ZYD4. Li et al., based on significant differences in metabolites of the marine red yeast *Sporobolomyces pararoseus*, found that most differentially expressed metabolites were enriched in amino acid metabolism, carbohydrate metabolism, and fat metabolism [54]. Ravishankar et al. found high salinity led the marine fungus *Cirrenalia pygmea* Kohl to increase the amino acid pool size [55]. In general, most free amino acids are considered to be osmolytes. It can be speculated that under high-salinity conditions, marine fungi maintain their normal physiological activities and increase their own amino acid production in order to cope with hypertonic conditions [56]. Amino acids serve as precursors for a variety of alkaloids, leading to changes in both the type and quantity of alkaloids produced. As a result, the 10% salinity PDB medium will yield a greater and different array of alkaloids.

In this study, the secondary metabolites of *A. terreus* C21-1 were compared in PDB medium with 3% salinity and 10% salinity, and there were significant differences in the yields of 11 alkaloids (VIP > 3). These alkaloids included quinoline and indole alkaloids derived from tryptophan, isoquinoline alkaloids from tyrosine, piperidine alkaloids from lysine, and pyrrolidine alkaloids from ornithine. It is hypothesized that the high-salt conditions may have led to an increase in amino acid production by activating normally silenced genes in *A. terreus* C21-1. Additionally, the abundance of precursor substances may have activated previously silent genes, resulting in increased production of certain alkaloids that are not typically synthesized under normal conditions.

In summary, different culture mediums, especially PDB with 10% salinity, significantly influence the metabolite expression and growth of *A. terreus* C21-1, as reflected by TLC, HPLC, morphological observation, and untargeted metabolomics. Multi-approach-assisted FBMN analysis indicated that alkaloids, which might be produced under high salt stress, contributed largely to the differences.

## 4. Materials and Methods

### 4.1. Materials and Chemicals

*A*. *terreus* C21-1 was isolated from the Xuwen Coral Reserve in Zhanjiang and was preserved in the Guangdong Provincial Microbial Culture Collection with deposit number GDMCC No. 62180. *Bacillus subtilis* MCCC 1A03710 was obtained from the China Marine Microbial Culture Collection, *Pseudomonas aeruginosa* from the American Type Culture Collection with ATCC number 9027, *Vibrio parahaemolyticus* was provided by Professor Wen Chongqing of Fisheries College at Guangdong Ocean University, *Vibrio alginolyticus* and *Shewanella putrefaciens* were supplied by Professor Liu Ying from the School of Food Science and Technology at Guangdong Ocean University, and MRSA A7983 was gifted by Professor Yu Zhijun from Dalian Friendship Hospital.

AChE (C3389, Sigma, Saint Louis, MO, USA), Acetylthiocholine iodide (DA0048, Sigma, Saint Louis, MO, USA), 1,1-Diphenyl-2-picrylhydrazyl (D9132, Sigma, Saint Louis, MO, USA), Bovine serum albumin (A1933, Sigma, Saint Louis, MO, USA), 96-well UV plate (Model 3635, Corning, Kennebunk, ME, USA), Sea crystal (Xuwen Hailong Marine biological preparations factory, Zhangjiang, China), Sucrose (Beijing Aoboxing Biotechnology Co., LTD, Beijing, China), Malt extract (Beijing Aoboxing Biotechnology Co., LTD, Beijing, China), peptone (Beijing Aoboxing Biotechnology Co., LTD, Beijing, China). All organic mobile-phase solvents used for LC-MS were from Merck (Darmstadt, Germany). All other reagents were of analytical purity (Guanghua Technology Co., LTD, Guangzhou, China).

An Agilent 1200 high-performance liquid chromatography and a Thermo Orbitrap Fusion LUMOS Tribrid liquid chromatography–mass spectrometer (Orbitrap LC-MS/MS, Thermo Fisher Scientific, Waltham, MA, USA) were used to analyze the samples. A 96-well microplate reader (Bio-Tek Epoch 2, Bio Tek Instruments, Winooski, VT, USA) was used for spectrophotometric measurements. Biosafety cabinet (BSC-1300 II A2, Boxun Industrial Co., LTD, Shanghai, China), Rotary evaporator (SB-1300, Ailang Instrument Co., LTD, Shanghai, China), Multifunctional UV transmission reflectometer (WFH-201 B, Jingke Industrial Co., LTD, Shanghai, China), Automatic autoclave (IRM-100, IRM Technology Co., LTD, Frankfurt, Germany).

### 4.2. Fermentation and Extraction for OSMAC Approach

*A. terreus* C21-1 was activated in PDA medium and cultured on a shaker for 3–4 days. The spore inoculum was then inoculated into six different media (0.3 PDB, 3 PDB, 10 PDB, 0.3 BRM, 0.3 FRM, and 0.3 NRM). Quantities of 0.3 PDB (potato extract 22.5 g/L, dextrose 2.5 g/L, sea salt 3 g/L), 3 PDB (potato extract 22.5 g/L, dextrose 2.5 g/L, sea salt 30 g/L), 10 PDB (potato extract 22.5 g/L, dextrose 2.5 g/L, sea salt 100 g/L). 0.3 BRM (brown rice 50 g/bottle, water 60 mL/bottle, sea salt 0.18 g/bottle), 0.3 FRM (fragrant rice 50 g, water 60 mL/bottle, sea salt 0.18 g/bottle), and 0.3 NRM (northeast rice 50 g, water 60 mL/bottle, sea salt 0.18 g/bottle) were cultivated at room temperature for 28 days. At the end of the fermentation, the extraction was repeated 3 times with ethyl acetate.

### 4.3. TLC and HPLC Analyses

All samples were prepared as methanol solutions with a concentration of 10 mg/mL for TLC and HPLC analysis. The mobile phase consisted of *n*-hexane: ethyl acetate in a ratio of 3:2 (*v*/*v*), using silica gel 60 F_254_ plates produced by Merck, with a volume of 10 µL. The plates were observed under UV lights at the wavelengths of 254 and 365 nm.

For the analysis, a Kinetex C18 100 Å reversed-phase column (100 × 4.60 mm, 5 μm) was utilized, with a sample injection volume of 5.0 µL. Water was used as mobile phase A, while mobile phase B consisted of methanol. The gradient elution conditions were as follows: starting at 50% B and increasing to 100% B over the course of 15 min, maintaining at 100% B for an additional five minutes, all at a flow rate of 0.6 mL/min.

### 4.4. AChE Inhibitory Activity Assay

The AChE inhibition activities were measured in 96-well plates using an optimized colorimetric method described previously [25]. All tests were carried out in three replicates. Donepezil hydrochloride was taken as the positive control.

### 4.5. DPPH Free Radical Scavenging Assay

An optimized colorimetric method described previously was used to evaluate the DPPH free radical scavenging activities of six crude extracts [57]. All tests were measured in three replicates. Ascorbic acid was taken as a positive control.

### 4.6. Antibacterial Assay

Antibacterial activities were measured against six indicator strains using the bilayer agar plate–Oxford cup method [58]. Ampicillin sodium was taken as a positive control.

### 4.7. LC-MS Analyses

Each crude extract was prepared to a concentration of 50 µg/mL using mass spectrometry-grade methanol and pre-treated on an Agilent SPE column prior to analysis [59].

The LC-MS/MS analysis was performed on a Waters Acquity UHPLC-DAD-Xevo G2-XS Q-Tof liquid chromatography–mass spectrometer, using a Waters ACQUITY UPLC BEH RP18 (2.1 × 50 mm, 1.7 µm) column. The injection volume was 1.0 µL, with 0.1% formic acid in water used as mobile phase A and acetonitrile as mobile phase B. The gradient elution conditions were set at 30–80% B over the first 8 min at a flow rate of 0.3 mL/min. The MS scanning range was *m*/*z* 50–2000 in positive ion mode, with the following parameters: ion source temperature at 120 °C, capillary at 2 KV, sampling cone at 40 V, source offset at 80 V, desolventization temperature at 450 °C, and cone gas flow rate of 0.5 mL/min. Mixtures of all extracts of the same concentration were prepared as quality control (QC) substances and injected periodically throughout the sequence for assessment during sample preparation, data collection, and data preprocessing steps.

### 4.8. Data Processing and Statistical Analysis

The following procedure was used: Process the LC-MS raw files using MS-DIAL and export the generated files in *.mgf format. Create a molecular network with FBMN and visualize the network using Cytoscape 3.7.1. Export the compound list to a CSV file using Cytoscape. The data underwent Pareto scaling and were subsequently analyzed using multivariate statistical methods, including PCA and OPLS-DA. To evaluate the robustness of the models, we applied 10-fold cross-validation along with response permutation tests (*n* = 200) for differentiating between liquid and solid media, as well as between 0.3% and 10% salinity conditions. The importance of each variable was quantified by computing variable importance in projection (VIP) scores within the OPLS-DA framework. Variables with VIP scores greater than 3 were considered to represent significantly differentiated metabolites. For these analyses, PCA was conducted using the R packages FactoMineR [60] and factoextra (https://cran.r-project.org/web/packages/factoextra/), accessed on 26 July 2024. while OPLS-DA was performed using the “ropls” package [61]. All analyses were carried out in R 4.1.2.

### 4.9. FBMN Analysis

The standard pipeline for FBMN molecular networking was executed in accordance with previous reports [26].The data visualization was carried out with Cytoscape 3.7.2 software.

### 4.10. Annotation of MS Features

The MS/MS data were initially fully annotated using the MS-Finder platform. Subsequently, the unannotated features were further inferred from the correlation and relative molecular weight in the GNPS [62] network map and queried for their structural presence using the SciFinder database. Finally, the consistency of fungal annotations’ classification and combination was further confirmed through a literature search.

## Figures and Tables

**Table 1:** The AChE inhibitory activities of G1–G6.

Sample Number	IC_50_/(μg/mL)	Inhibition (%) ^b^
G1	5.2 ± 0.6	72.6 ± 0.5
G2	32.4 ± 1.2	15.0 ± 1.1
G3	52.3 ± 0.9	12.3 ± 1.2
G4	0.5 ± 0.7	71.9 ± 0.3
G5	0.4 ± 0.8	73.2 ± 1.5
G6	0.7 ± 0.1	80.6 ± 1.4
Donepezil hydrochloride ^a^	0.2 ± 0.1	99.1 ± 2.0

^a^ means positive control, ^b^ means sample concentration was 1.56 μg/mL.

**Table 2:** The DPPH free radical scavenging activities of G1–G6.

Sample Number	EC_50_/(μg/mL)	Inhibition (%) ^b^
G1	57.2 ± 0.8	90.7 ± 1.2
G2	183.2 ± 0.6	76.0 ± 0.8
G3	352.3 ± 1.1	52.0 ± 1.2
G4	36.5 ± 0.9	91.7 ± 1.5
G5	31.7 ± 0.7	93.9 ± 0.6
G6	88.8 ± 1.2	90.1 ± 1.9
Ascorbic acid ^a^	7.2 ± 0.2	97.1 ± 1.1

^a^ means positive control, ^b^ means sample concentration was 0.5 mg/mL.

**Table 3:** Antimicrobial activities of G1–G6, measured using the Oxford cup method (dosage: 200 mL/well, concentration = 1 mg/mL, *n* = 4). Diameters of inhibition zones against indicator microbes (mm).

Sample Number	*MRSA*	*Pseud* *omonus* *aeruginosa*	*Vibr* *i* *o* *parah* *a* *emo* *lyticus*	*Vibr* *i* *o* *alginol* *yticus*	*Shewanella* *putrefaciens*	*Bacillus* *subtilis*
G1	14.0 ± 0.6	13.0 ± 0.3	13.0 ± 0.5	13.0 ± 0.2	10.0 ± 0.7	14.0 ± 0.2
G2	-	-	-	-	-	-
G3	-	-	-	-	-	-
G4	10.5 ± 0.2	13.0 ± 0.6	13.0 ± 0.5	15.5 ± 0.2	11.3 ± 0.3	13.5 ± 0.6
G5	14.0 ± 0.6	13.0 ± 0.7	13.0 ± 0.2	18.0 ± 0.5	13.0 ± 0.5	14.0 ± 0.7
G6	-	-	-	-	-	-
Ampicillin ^a^	14.0 ± 0.8	19.0 ± 0.5	24.0 ± 0.7	19.0 ± 0.6	16.0 ± 0.9	18.0 ± 0.2

^a^ means positive control, - means no activity or very weak activity.

## Data Availability

Data are contained within the article.

## Supplementary Materials

The following are available online at https://www.mdpi.com/article/10.3390/ijms251910544/s1.
