# Supplementary material for: Untargeted Metabolomics Approach for the Discovery of Salinity-Related Alkaloids in a Stony Coral-Derived Fungus Aspergillus terreus
Source: Int J Mol Sci. 2024 Sep 30;25(19):10544. doi: 10.3390/ijms251910544 (PMC11476925; doi:10.3390/ijms251910544)
Supplement: Supplementary file 1 [file ijms-25-10544-s001.zip › Supplement.pdf]

# Supporting Information

## Untargeted Metabolomics Approach for the Discovery of Salinity-Related Alkaloids in a Stony Coral-Derived Fungus *Aspergillus terreus*

**Yayue Liu[1,2,3], Li Wang[1], Yunkai Feng[1], Qingnan Liao[1], Xiaoling Lei[1,2,3], Xueqiong Hu[1], Longjian Zhou[1,2,3*], Yi Zhang[1,2,3*]**

[1] Guangdong Provincial Key Laboratory of Aquatic Product Processing and Safety, Guangdong Provincial Engineering Laboratory for Marine Biological Products, Guangdong Provincial Center for Modern Agricultural Scientific Innovation, Shenzhen Institute of Guangdong Ocean University, Zhanjiang Municipal Key Laboratory of Marine Drugsand Nutrition for Brain Health, Research Institute for Marine Drugs and Nutrition, College of Food Science and Technology, Guangdong Ocean University, Zhanjiang 524088, China; yayue_liu@163.com (Y.L.); wangli991003@163.com (L.W.); 13582945747@163.com (Y.F); 17854225126@163.com (Q.L.); leixl-19@126.com (X.L.); hxw247@163.com (X.H.)

[2] Southern Marine Science and Engineering Guangdong Laboratory (Zhanjiang), Zhanjiang 524088, China

[3] Collaborative Innovation Center of Seafood Deep Processing, Dalian Polytechnic University, Dalian 116034, China

* Correspondence: zhoulongjian@gdou.edu.cn & hubeizhangyi@163.com

## Tables of Contents

**Table S1.** Putative identification of characteristic ions highlighted in OPLS-DA S-plots in the liquid and soild culture medium.

**Table S2.** Putative identification of characteristic ions highlighted in OPLS-DA S-plots in the 0.3% salinity and 10% salinity PDB culture medium.

**Table S1.** Putative identification of characteristic ions highlighted in OPLS-DA S-plots in the liquid and soild culture medium.

| VIP | *m/z* | $t_R$(min) | Observed Features (Ionic Species) | Molecular Formula for M | Putative Annotation |
|---|---|---|---|---|---|
| 10.94 | 256.2578 | 7.827 | $[M+H]^+$ | - | - |
| 10.63 | 282.2785 | 8.265 | $[M+H]^+$ | $C_{18}H_{35}NO$ | 9-Octadecenamide |
| 7.94 | 438.1777 | 3.921 | $[M+H]^+$ | $C_{22}H_{26}F_3N_3OS$ | Fluphenazine |
| 6.95 | 500.3941 | 6.36 | $[M+H]^+$ | - | - |
| 6.64 | 280.2579 | 6.847 | $[M+H]^+$ | - | - |
| 6.44 | 301.1377 | 5.882 | $[M+H]^+$ | $C_{18}H_{20}O_4$ | Angoletin |
| 6.36 | 396.7977 | 1.074 | $[M+H]^+$ | - | - |
| 6.28 | 498.3773 | 5.674 | $[M+H]^+$ | - | - |
| 6.25 | 416.1958 | 3.921 | $[M+H]^+$ | $C_{23}H_{29}NO_6$ | (3*S*,11*R*,11a*R*)-3'-Methoxy-4',9-dimethyl-3beta-[[(2*S*,4*S*)-4-methyl-5-oxotetrahydrofuran]-2-yl]-2,3,5,6,7,8-hexahydrospiro[1*H*-cyclopenta[b]pyrrolo[1,2-a]azepine-11(10*H*),2'(5'*H*)-furan]-5',10-dione |
| 6.19 | 679.5161 | 0.944 | $[M+H]^+$ | - | - |
| 6.03 | 454.1718 | 3.435 | $[M+H]^+$ | $C_{21}H_{24}O_{10}$ | Phlorizine |
| 5.88 | 927.6724 | 1.236 | $[M+H]^+$ | - | - |
| 5.86 | 340.2553 | 0.945 | $[M+H]^+$ | - | - |
| 5.83 | 701.4984 | 0.946 | $[M+H]^+$ | - | - |
| 5.82 | 298.1503 | 3.321 | $[M+H]^+$ | $C_{15}H_{21}N_3O_2$ | 1,3a,8-Trimethyl-1,2,3,3a,8,8a-hexahydropyrrolo[2,3-b]indol-5-yl methylcarbamate |
| 5.56 | 283.1402 | 4.951 | $[M+H]^+$ | $C_{12}H_{18}N_4O_4$ | - |
| 5.49 | 316.1610 | 3.321 | $[M+H]^+$ | $C_{18}H_{21}NO_4$ | Percodan |
| 5.18 | 507.2708 | 7.002 | $[M+H]^+$ | $C_{25}H_{38}N_4O_5S$ | 1-[1-[3-Methyl-2-[(4-methylphenyl)sulfonylamino]pentanoyl]piperidine-4-carbonyl]piperidine-4-carboxamide |
| 4.93 | 453.3412 | 1.235 | $[M+2H]^{2+}$ | $C_{30}H_{44}O_3$ | [(9*S*,14*S*,17*S*)-10,13-dimethyl-3-oxo-6,7,8,9,11,12,14,15,16,17-decahydrocyclopenta[a]phenanthren-17-yl] undec-10-enoate |
| 4.74 | 259.1595 | 2.095 | $[M+Na]^+$ | $C_{15}H_{24}O_2$ | Curdione |
| 4.74 | 265.2471 | 8.268 | $[M+H]^+$ | - | - |
| 4.42 | 245.1438 | 1.317 | $[M+H]^+$ | $C_{12}H_{20}O_5$ | - |
| 4.38 | 210.0698 | 3.652 | $[M+H]^+$ | $C_{10}H_{11}NO_4$ | 2-Methylidene-3-oxo-4*A*,7,8,8*A*-tetrahydro-4*H*-1,4-benzoxazine-7-carboxylic acid |
| 4.31 | 274.2708 | 4.193 | $[M+H]^+$ | $C_{16}H_{32}O_2$ | 14-Methylpentadecanoic acid |
| 4.27 | 475.3232 | 0.758 | $[M+H]^+$ | - | - |
| 4.25 | 905.6905 | 1.225 | $[M+H]^+$ | - | - |
| 3.93 | 212.052 | 4.951 | $[M+H]^+$ | $C_9H_9NO_5$ | DIMBOA |
| 3.86 | 566.4279 | 0.835 | $[M+H]^+$ | - | - |
| 3.85 | 520.3391 | 5.252 | $[M+H]^+$ | $C_{26}H_{5}0NO_7P$ | (2-Hydroxy-3-octadeca-9,12-dienoyloxypropyl) 2-(trimethylazaniumyl)ethyl phosphate |
| 3.73 | 318.2961 | 4.233 | $[M+H]^+$ | $C_{18}H_{39}NO_3$ | 2-Aminooctadecane-1,3,4-triol |
| 3.72 | 588.4104 | 0.834 | $[M+H]^+$ | - | - |

| | | | | | |
|---|---|---|---|---|---|
| 3.68 | 427.2425 | 5.483 | $[M+Na]^+$ | $C_{24}H_{36}O_5$ | Monacolin K |
| 3.54 | 247.2363 | 8.266 | $[M+H]^+$ | - | - |
| 3.48 | 263.2311 | 6.846 | $[M+H]^+$ | $C_{18}H_{30}O$ | - |
| 3.37 | 227.1331 | 0.736 | $[M+H]^+$ | $C_{12}H_{18}O_4$ | - |
| 3.36 | 236.1434 | 6.357 | $[M+H]^+$ | - | - |
| 3.26 | 285.0707 | 3.984 | $[M+H]^+$ | $C_{16}H_{12}O_5$ | Negletein |
| 3.13 | 236.1431 | 5.673 | $[M+H]^+$ | - | - |
| 3.10 | 317.2056 | 5.602 | $[M+H]^+$ | $C_{20}H_{30}O_4$ | 6,18,19-Trihydroxytrachyloban-2-one |
| 3.07 | 529.2099 | 5.108 | $[M+Na]^+$ | $C_{32}H_{30}N_2O_4$ | (2-Benzamido-3-phenylpropyl) 2-benzamido-3-phenylpropanoate |
| 3.06 | 290.2640 | 4.262 | $[M+H]^+$ | - | - |
| 3.01 | 453.3409 | 0.757 | $[M+H]^+$ | $C_{30}H_{44}O_3$ | [(9*S*,14*S*,17*S*)-10,13-dimethyl-3-oxo-6,7,8,9,11,12,14,15,16,17-decahydrocyclopenta[a]phenanthren-17-yl] undec-10-enoate |

- means no compounds are commented

**Table S2**. Putative identification of characteristic ions highlighted in OPLS-DA S-plots in the 0.3% salinity and 10% salinity PDB culture medium.

| VIP | m/z | Rt (min) | Observed Features (Ionic Species) | Molecular Formula for M | Putative Annotation |
|---|---|---|---|---|---|
| 9.19 | 438.1777 | 3.921 | $[M+H]^+$ | $C_{22}H_{26}F_3N_3OS$ | Fluphenazine |
| 8.15 | 416.1958 | 3.922 | $[M+H]^+$ | $C_{23}H_{29}NO_6$ | 4'-methoxy-3',11-dimethyl-4-(4-methyl-5-oxooxolan-2-yl)spiro[5-azatricyclo[8.3.0.01,5]tridec-10-ene-13,5'-furan]-2',12-dione |
| 8.14 | 283.1402 | 4.951 | $[M+H]^+$ | $C_{12}H_{18}N_4O_4$ | - |
| 7.30 | 454.1718 | 3.435 | $[M+H]^+$ | $C_{24}H_{23}NO_8$ | 1-[2-[[2-[(2,5-dimethoxyphenyl)methylidene]-3-oxo-1-benzofuran-6-yl]oxy]acetyl]pyrrolidine-2-carboxylic acid |
| 7.22 | 701.4984 | 0.946 | - | - | - |
| 7.10 | 298.1503 | 3.321 | $[M+H]^+$ | $C_{15}H_{21}N_3O_2$ | ([(3a*R*,8b*S*)-3,4,8b-trimethyl-2,3a-dihydro-1*H*-pyrrolo [2,3-b]indol-7-yl] *N*-methylcarbamate) |
| 7.09 | 274.2708 | 4.193 | $[M+H]^+$ | $C_{16}H_{32}O_2$ | 14-Methylpentadecanoic acid |
| 6.99 | 301.1377 | 5.882 | $[M+H]^+$ | $C_{18}H_{20}O_4$ | Angoletin |
| 6.64 | 259.1595 | 2.095 | $[M+Na]^+$ | $C_{15}H_{24}O_2$ | Curdione |
| 6.53 | 316.161 | 3.321 | $[M+H]^+$ | $C_{18}H_{21}NO_4$ | Percodan |
| 6.17 | 318.2962 | 4.233 | $[M+H]^+$ | $C_{18}H_{39}NO_3$ | 2-Aminooctadecane-1,3,4-triol |
| 6.15 | 245.1438 | 1.317 | $[M+H]^+$ | $C_{12}H_{20}O_5$ | - |
| 6.02 | 588.4104 | 0.834 | $[M+H]^+$ | - | - |
| 5.75 | 212.052 | 4.951 | $[M+H]^+$ | $C_9H_9NO_5$ | DIMBOA |
| 5.55 | 679.5161 | 0.944 | $[M+H]^+$ | - | - |
| 5.11 | 520.3391 | 5.252 | $[M+H]^+$ | $C_{26}H_{50}NO_7P$ | (2-hydroxy-3-octadeca-9,12-dienoyloxypropyl) 2-(trimethylazaniumyl)ethyl phosphate |
| 4.96 | 210.0698 | 3.652 | $[M+H]^+$ | $C_{10}H_{11}NO_4$ | 2-methylidene-3-oxo-4a,7,8,8a-tetrahydro-4*H*-1,4-benzoxazine-7-carboxylic acid |
| 4.61 | 500.3941 | 6.36 | $[M+H]^+$ | - | - |
| 4.54 | 427.2425 | 5.483 | $[M+Na]^+$ | $C_{24}H_{36}O_5$ | (2-hydroxy-3-octadeca-9,12-dienoyloxypropyl) 2-(trimethylazaniumyl)ethyl phosphate |
| 4.47 | 459.2697 | 5.602 | $[M+H]^+$ | $C_{27}H_{38}O_6$ | 3,7-dihydroxy-4,4,10,13,14-pentamethyl-17-(2-methyl-5-oxooxolan-2-yl)-2,3,5,6,7,12,16,17-octahydro-1*H*-cyclopenta[a]phenanthrene-11,15-dione |
| 4.46 | 566.4279 | 0.835 | $[M+H]^+$ | - | - |
| 4.46 | 529.2099 | 5.108 | $[M+Na]^+$ | $C_{32}H_{30}N_2O_4$ | (2-benzamido-3-phenylpropyl) 2-benzamido-3-phenylpropanoate |
| 4.39 | 282.2785 | 8.265 | $[M+H]^+$ | $C_{18}H_{35}NO$ | 9-Octadecenamide |
| 4.36 | 396.7977 | 1.074 | $[M+H]^+$ | - | - |
| 4.18 | 475.3232 | 0.758 | $[M+H]^+$ | - | - |
| 4.16 | 317.2058 | 5.602 | $[M+H]^+$ | $C_{20}H_{30}O_4$ | 6,18,19-Trihydroxytrachyloban-2-one |
| 4.15 | 340.2553 | 0.945 | $[M+H]^+$ | - | - |
| 3.81 | 253.0916 | 4.951 | $[M+H]^+$ | $C_{16}H_{12}O_3$ | 3-(4-methoxyphenyl)isochromen-1-one |
| 3.69 | 306.1192 | 3.92 | $[M+H]^+$ | $C_{17}H_{17}NO_3$ | Cinnamamide, p-hydroxy-*N*-(p-hydroxyphenethyl)- |
| 3.54 | 195.0591 | 4.37 | $[M+H]^+$ | $C_{10}H_{10}O_4$ | 3-(3-hydroxy-4-methoxyphenyl)prop-2-enoic acid |
| 3.44 | 291.0816 | 4.372 | $[M+H]^+$ | $C_{15}H_{14}O_6$ | Epicatechin |

| | | | | | |
|---|---|---|---|---|---|
| 3.43 | 927.6724 | 1.236 | $[M+H]^+$ | - | - |
| 3.38 | 479.1274 | 2.903 | $[M+Na]^+$ | $C_{22}H_{21}FN_4O_6$ | 5-(diaminomethylideneamino)-2-[[3-(4-fluorophenyl)-7-hydroxy-4-oxochromene-2-carbonyl]amino]pentanoic acid |
| 3.38 | 285.1795 | 4.969 | $[M+H]^+$ | $C_{19}H_{24}O_2$ | 10,13-dimethyl-7,8,9,11,12,14,15,16-octahydro-6*H*-cyclopenta[a]phenanthrene-3,17-dione |
| 3.36 | 471.2361 | 4.581 | $[M+H]^+$ | $C_{25}H_{36}O_7$ | (2-Hydroxy-6,10-dimethyl-3-propan-2-yl-11-oxabicyclo[8.1.0]undec-6-en-4-yl) 3,4,5-trimethoxybenzoate |
| 3.26 | 453.3409 | 0.757 | $[M+H]^+$ | $C_{30}H_{44}O_3$ | [(9*S*,14*S*,17*S*)-10,13-dimethyl-3-oxo-6,7,8,9,11,12,14,15,16,17-decahydrocyclopenta[a]phenanthren-17-yl] undec-10-enoate |
| 3.24 | 130.0583 | 3.925 | $[M+H-H_2O]^+$ | $C_9H_9NO$ | Indole-3-carbinol |
| 3.24 | 284.289 | 7.68 | $[M+H]^+$ | $C_{18}H_{37}NO$ | Octadecanamide |
| 3.20 | 339.227 | 6.775 | $[M+H]^+$ | $C_{21}H_{32}O_2$ | 5-Pentadeca-8,11-dienylbenzene-1,3-diol |
| 3.16 | 488.1417 | 3.652 | $[M+H]^+$ | - | - |
| 3.05 | 304.1034 | 3.438 | $[M+H]^+$ | $C_{10}H_{18}NNaO_8$ | - |
| 3.02 | 706.4579 | 2.635 | $[M+H]^+$ | $C_{39}H_{63}NO_{10}$ | beta1-Chaconine |
| 3.01 | 374.1834 | 4.363 | $[M+H]^+$ | $C_{23}H_{23}N_3O_2$ | 3-(3,4-Dimethoxyphenyl)-6-(3-propan-2-ylphenyl)-[1,2,4]triazolo[4,3-a]pyridine |

- means no compounds are commented
